# Luminal epithelium in endometrial fragments affects their vascularization, growth and morphological development into endometriosis-like lesions in mice

**DOI:** 10.1242/dmm.013664

**Published:** 2013-11-28

**Authors:** Dilu Feng, Michael D. Menger, Hongbo Wang, Matthias W. Laschke

**Affiliations:** 1 Institute for Clinical & Experimental Surgery, University of Saarland, 66421 Homburg/Saar, Germany.; 2 Department of Obstetrics and Gynecology, Union Hospital, Tongji Medical College, Huazhong University of Science and Technology, Wuhan 430022, China.

**Keywords:** Endometriosis, Endometriotic lesion, Luminal epithelium, Vascularization, Angiogenesis, Morphology, Intravital fluorescence microscopy, Dorsal skinfold chamber

## Abstract

In endometriosis research, endometriosis-like lesions are usually induced in rodents by transplantation of isolated endometrial tissue fragments to ectopic sites. In the present study, we investigated whether this approach is affected by the cellular composition of the grafts. For this purpose, endometrial tissue fragments covered with luminal epithelium (LE^+^) and without luminal epithelium (LE^−^) were transplanted from transgenic green-fluorescent-protein-positive (GFP^+^) donor mice into the dorsal skinfold chamber of GFP^−^ wild-type recipient animals to analyze their vascularization, growth and morphology by means of repetitive intravital fluorescence microscopy, histology and immunohistochemistry during a 14-day observation period. LE^−^ fragments developed into typical endometriosis-like lesions with cyst-like dilated endometrial glands and a well-vascularized endometrial stroma. In contrast, LE^+^ fragments exhibited a polypoid morphology and a significantly reduced blood perfusion after engraftment, because the luminal epithelium prevented the vascular interconnection with the microvasculature of the surrounding host tissue. This was associated with a markedly decreased growth rate of LE^+^ lesions compared with LE^−^ lesions. In addition, we found that many GFP^+^ microvessels grew outside the LE^−^ lesions and developed interconnections to the host microvasculature, indicating that inosculation is an important mechanism in the vascularization process of endometriosis-like lesions. Our findings demonstrate that the luminal epithelium crucially affects the vascularization, growth and morphology of endometriosis-like lesions. Therefore, it is of major importance to standardize the cellular composition of endometrial grafts in order to increase the validity and reliability of pre-clinical rodent studies in endometriosis research.

## INTRODUCTION

Endometriosis, which is defined as the presence of ectopic endometrial tissue in the form of endometriotic lesions outside the uterine cavity, is one of the most frequent gynecological diseases (Guo and Wang, 2006). Due to its pain symptoms, chronic progression and high recurrence rates, endometriosis is often associated with a severely altered quality of the patients’ private and professional life (Gao et al., 2006; Stratton and Berkley, 2011). Accordingly, there is a substantial need for novel therapeutic strategies that are better tolerated and more efficient than the currently applied pharmacological and surgical approaches. For this purpose, it is necessary to gain better insights into the pathogenesis of endometriosis, which is still sparsely understood. Various theories have been postulated during the last decades (Viganò et al., 2004; Leyendecker et al., 2009; Maruyama and Yoshimura, 2012). The most widely accepted one, however, is still Sampson’s theory of retrograde menstruation of endometrial tissue fragments into the peritoneal cavity, where they attach to the peritoneum and develop into vascularized endometriotic lesions (Sampson, 1927).

In endometriosis research, genetically well-defined rodent models are an essential tool in early stages of drug testing (Laschke and Menger, 2012). Moreover, they are highly suitable for unraveling the complex molecular and cellular mechanisms that contribute to the onset and progression of the disease (Tirado-González et al., 2010). However, in contrast to humans and non-human primates, rodents do not menstruate and, thus, do not develop endometriosis spontaneously. Hence, it is necessary to induce endometriosis-like lesions in these animals iatrogenically, which is usually achieved by transplantation of isolated endometrial tissue fragments to ectopic sites (Grümmer, 2006). A major problem of this approach is the heterogeneous composition of the transplanted tissue, which consists of luminal epithelium, glandular epithelium and fibroblastic stroma. Accordingly, differing fractions of these cellular components in individual grafts could markedly affect the standardized induction of endometriosis-like lesions. In particular, the luminal epithelium of the endometrium with its anti-adhesive properties is well known to act as a physical and immunological barrier (Gipson et al., 2008; Ochiel et al., 2008). Therefore, we speculated that the existence of this luminal epithelium in isolated endometrial tissue fragments might crucially affect their vascularization and morphological development into endometriosis-like lesions after transplantation to an ectopic site.

## RESULTS

**Fig. 1.**
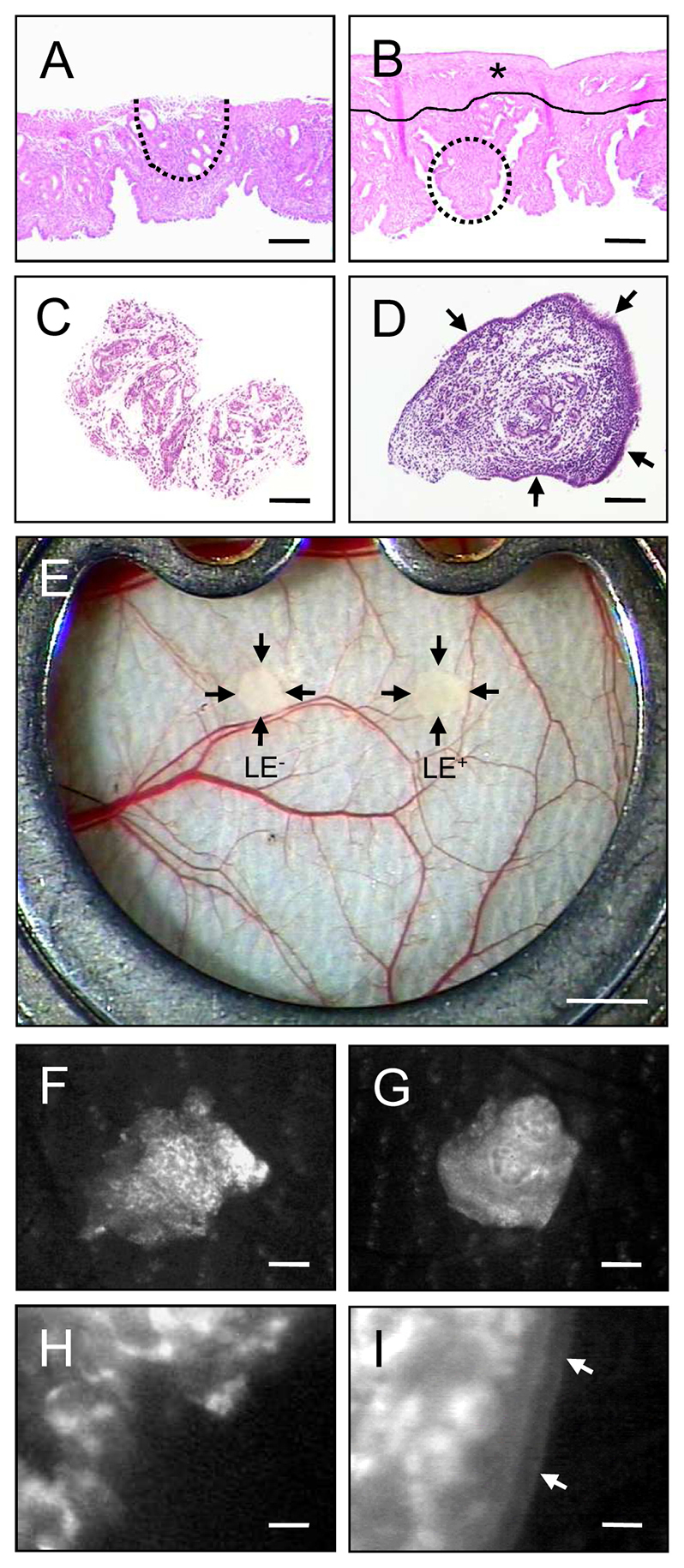
**Dorsal skinfold chamber model of endometriosis.** (A,B) H&E-stained sections of the longitudinally opened uterine horns from a C57BL/6-TgN(ACTB-EGFP)1Osb/J donor mouse for the isolation of a LE^−^ fragment (A, dotted line) and a LE^+^ fragment (B, dotted line). The LE^+^ fragment was excised from the luminal side of the endometrium of an intact uterine horn, which exhibits a layer of myometrium with the underlying perimetrium (B, asterisk). For the isolation of the LE^−^ fragment, this layer is first removed (compare A with B) and the fragment is then excised from the exposed basal endometrium (A). (C,D) H&E-stained sections of a LE^−^ fragment (C) and a LE^+^ fragment (D) directly after the isolation procedure. The luminal epithelium covering the LE^+^ fragment is clearly visible (D, arrows). (E) Observation window of the dorsal skinfold chamber of a C57BL/6 wild-type mouse directly after transplantation of a LE^−^ fragment and a LE^+^ fragment onto the host striated muscle tissue (transplants are indicated by arrows). (F–I) Intravital fluorescent microscopic images of a LE^−^ fragment (F,H) and a LE^+^ fragment (G,I) directly after transplantation into the dorsal skinfold chamber. The fragments can be easily detected in blue light epi-illumination due to their GFP signal. The luminal epithelium of the LE^+^ fragment is clearly visible at higher magnification (I, arrows). Scale bars: 170 μm (A,B), 90 μm (C,D), 1.3 cm (E), 220 μm (F,G), 20 μm (H,I).

To investigate the effect of the luminal epithelium on the vascularization and morphological development of endometrial tissue fragments into endometriosis-like lesions, we used the dorsal skinfold chamber model. This model allows for the repetitive *in vivo* analysis of blood vessel formation in endometriosis-like lesions by means of intravital fluorescence microscopy (Laschke et al., 2005; Laschke and Menger, 2007). The endometrial tissue fragments were isolated from the uterine horns of transgenic C57BL/6-TgN(ACTB-EGFP)1Osb/J mice. In these mice, with an enhanced green fluorescent protein (GFP) cDNA under the control of a chicken β-actin promoter and cytomegalovirus enhancer, all of the tissues (with exception of erythrocytes and hair) exhibit a green fluorescence under blue light excitation (Okabe et al., 1997). For the generation of endometrial tissue fragments lacking a luminal epithelium (LE^−^ fragments), the myometrium with the underlying perimetrium was carefully removed from one uterine horn. Subsequently, small LE^−^ fragments were excised from the exposed basal endometrium (Fig. 1A,C). From the second uterine horn, endometrial tissue fragments covered with a luminal epithelium (LE^+^ fragments) were isolated from the luminal side of the endometrium (Fig. 1B,D). Both fragment types exhibited a comparable initial size of 0.7–0.9 mm^2^.

**TRANSLATIONAL IMPACT**

**Clinical issue**

Endometriosis, one of the most frequently occurring female gynecological disorders, is often associated with a severely reduced quality of life because of its association with heavy and painful menstruation, abdominal pain and fertility problems. Accordingly, there is an urgent need for the development of efficient treatment strategies. For this purpose, genetically well-defined rodent models are important tools because they provide new insights into the complex pathophysiology of the disease and can be used in the early stages of drug testing. In the existing rodent models for this disorder, endometriosis-like lesions are usually induced by transplantation of isolated endometrial tissue fragments to ectopic sites. This approach, however, might be markedly affected by the cellular composition of the used fragments, potentially compromising the validity and reliability of pre-clinical endometriosis studies.

**Results**

In the present study, the authors investigate how the existence of luminal epithelium in isolated endometrial tissue fragments affects their vascularization and morphological development into endometriosis-like lesions. Using the dorsal skinfold chamber model (a system for the *in vivo* analysis of biomaterial implants), they demonstrate that fragments without luminal epithelium develop into typical endometriosis-like lesions with cyst-like dilated endometrial glands and a well-vascularized endometrial stroma. In contrast, fragments covered with a luminal epithelium exhibit a polypoid morphology as well as a significantly reduced blood perfusion and growth rate after engraftment. This is caused by the barrier function of the luminal epithelium, which prevents the interaction of the grafts with the surrounding host tissue.

**Implications and future directions**

This study reveals that absence of luminal epithelium of endometrial tissue fragments is crucial for their successful vascularization, growth and morphological development into endometriosis-like lesions in mice. Accordingly, it is of major importance to standardize endometrial grafts in order to increase the validity and reliability of pre-clinical animal studies in endometriosis research. In addition, all studies should provide a detailed description of the endometrial grafts used to enable a better comparability of the results from different laboratories. These efforts should strengthen research into endometriosis pathophysiology and advance the discovery of effective therapeutic strategies.

**Fig. 2.**
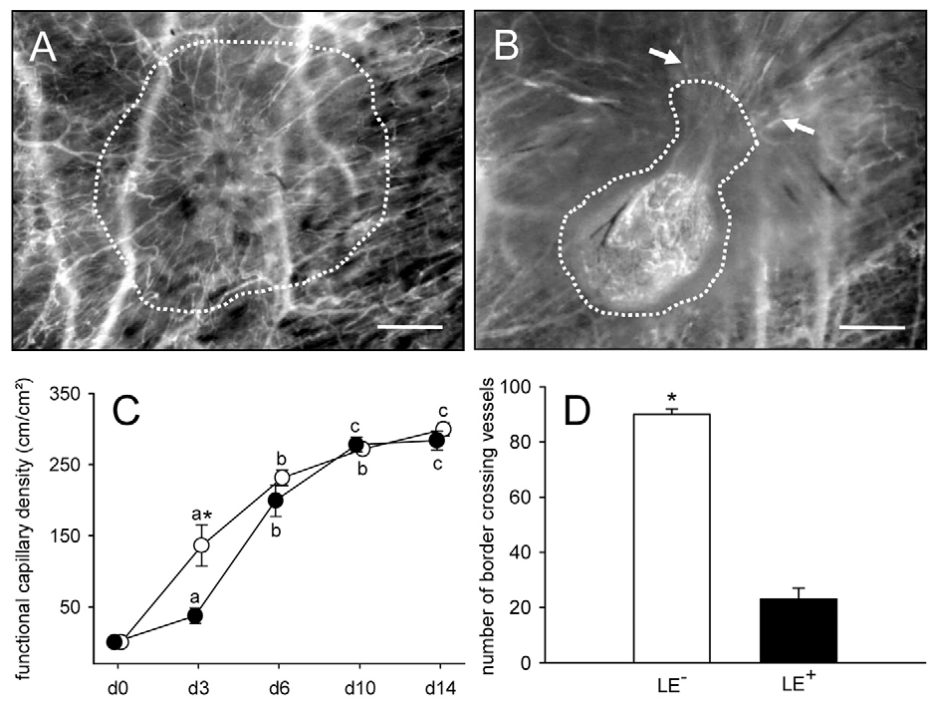
**Vascularization of transplanted endometrial tissue fragments.** (A,B) Intravital fluorescent microscopic images of a vascularized LE^−^ fragment (A, borders marked by dotted line) and a LE^+^ fragment (B, borders marked by dotted line) at day 14 after transplantation into the dorsal skinfold chamber of a C57BL/6 wild-type mouse. The microvascular network of the LE^−^ fragment exhibits many interconnections to the host microvasculature of the chamber tissue (A), whereas only a few microvessels pierce into the LE^+^ fragment (B, arrows). Blue light epi-illumination with contrast enhancement by intravascular staining of plasma with 5% FITC-labeled dextran 150,000 (i.v.). Scale bars: 290 μm. (C,D) Functional capillary density (cm/cm^2^) and number of border crossing vessels (at day 14) of LE^−^ fragments (white circles and bar graph, *n*=8) and LE^+^ fragments (black circles and bar graph, *n*=8) after transplantation into dorsal skinfold chambers of C57BL/6 wild-type mice, as assessed by intravital fluorescence microscopy and computer-assisted image analysis. Values are means ± s.e.m. ^a^*P*<0.05 versus day 0 within each individual group; ^b^*P*<0.05 versus days 0 and 3 within each individual group; ^c^*P*<0.05 versus days 0, 3 and 6 within each individual group; *P<0.05 versus LE^+^ fragments.

**Fig. 3.**
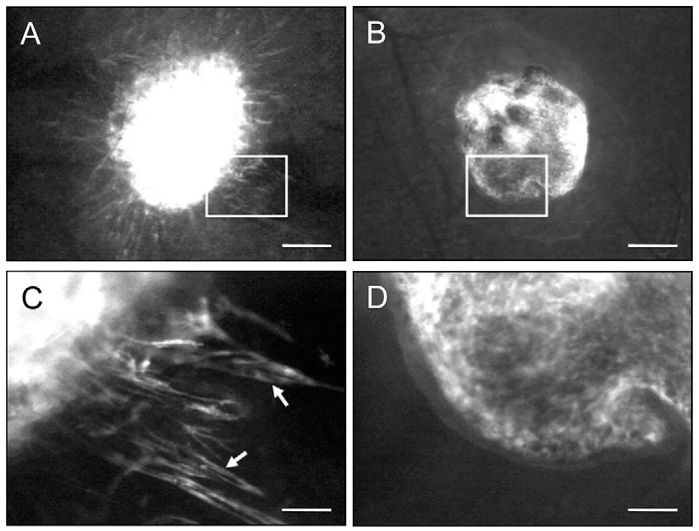
**Barrier function of the luminal epithelium during fragment vascularization.** (A,B) Intravital fluorescence microscopic images of a LE^−^ fragment (A) and a LE^+^ fragment (B) at day 6 after transplantation into the dorsal skinfold chamber of a C57BL/6 wild-type mouse. (C,D) Higher magnification images of windows in A and B, respectively. The detection of GFP in blue light epi-illumination reveals that many GFP^+^ microvessels (C, arrows) grow out of the LE^−^ fragment. By contrast, in the LE^+^ fragment microvessels do not pass the luminal epithelium and, thus, do not grow out of the graft (D). Scale bars: 310 μm (A,B), 90 μm (C,D).

Directly after transplantation into the dorsal skinfold chamber of C57BL/6 wild-type mice, the endometrial tissue fragments could easily be detected due to their GFP signal (Fig. 1E–I). LE^−^ fragments typically exhibited an irregular shape with a frayed border (Fig. 1F,H), whereas LE^+^ fragments were rounded and sharply separated from the surrounding host tissue by their luminal epithelium (Fig. 1G), which could be clearly visualized in higher magnification (Fig. 1I). During the time course of the experiment, both fragment types vascularized and finally exhibited dense microvascular networks with a comparable functional capillary density of ~300 cm/cm^2^ at day 14 (Fig. 2A–C). However, the vascularization process was found accelerated in LE^−^ fragments, as indicated by a significantly higher functional capillary density at day 3 compared with that of LE^+^ fragments (Fig. 2C). Moreover, the final morphology of the microvascular networks markedly differed between LE^−^ and LE^+^ fragments. Microvascular networks of LE^−^ fragments developed many interconnections to the host microvasculature of the chamber tissue (Fig. 2A,D), whereas only a few microvessels pierced into the LE^+^ fragments. Of interest, these few interconnecting microvessels were restricted to LE^+^ sites, which were not covered with luminal epithelium (Fig. 2B,D). By transplanting GFP^+^ endometrial tissue fragments into GFP^−^ recipient animals, we could further demonstrate that both LE^−^ and LE^+^ fragments still exhibited GFP^+^ microvessels at day 14 after transplantation. In LE^−^ fragments many GFP^+^ microvessels grew out of the grafts into the surrounding host tissue, where they developed interconnections to the GFP^−^ microvessels of the chamber tissue (Fig. 3A,C). In contrast, the GFP^+^ microvessels of LE^+^ fragments did not pass the luminal epithelium (Fig. 3B,D).

**Fig. 4.**
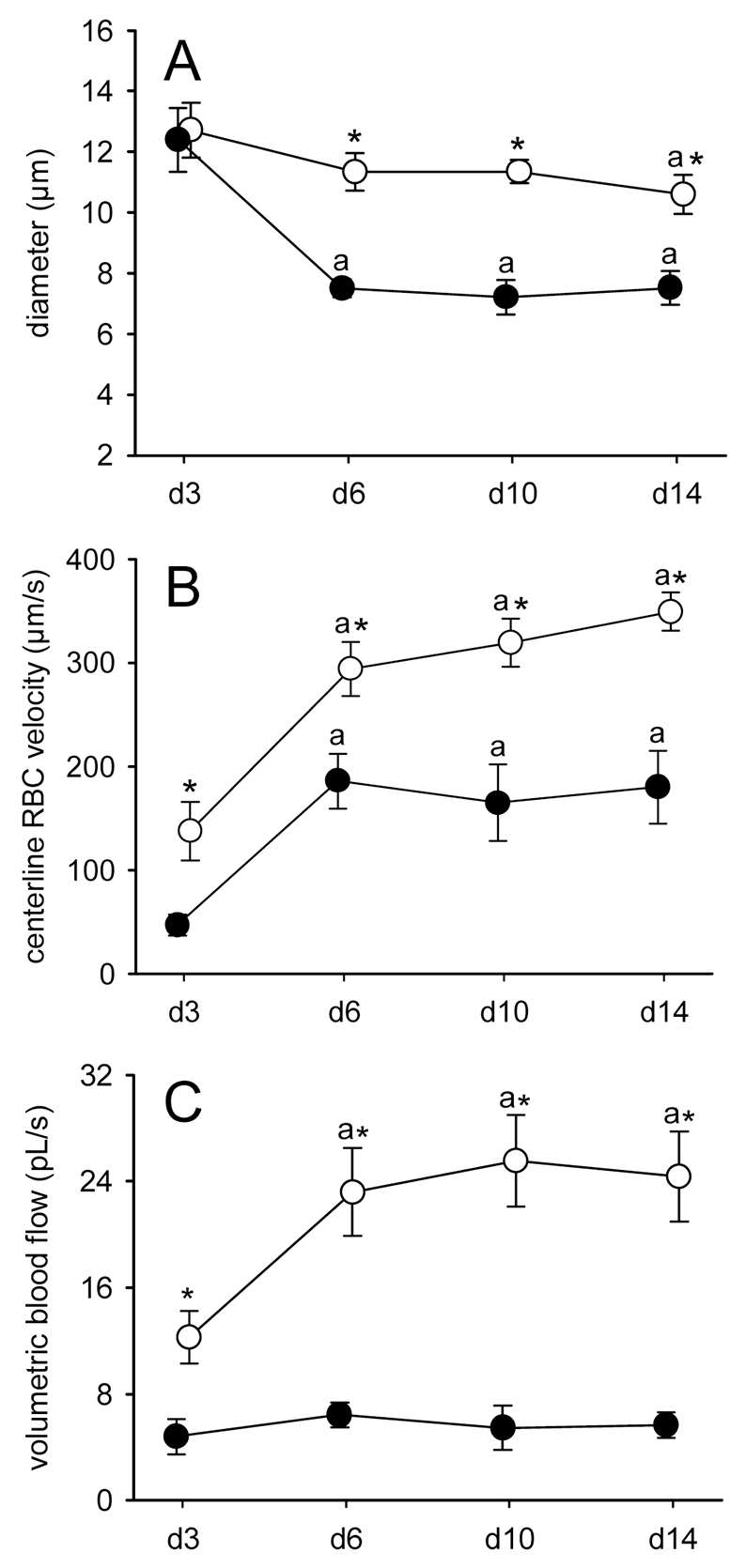
**Microhemodynamics of transplanted endometrial tissue fragments.** (A–C) Diameter (A), centerline RBC velocity (B) and volumetric blood flow (C) of microvessels in LE^−^ fragments (white circles, *n*=8) and LE^+^ fragments (black circles, *n*=8) after transplantation into dorsal skinfold chambers of C57BL/6 wild-type mice, as assessed by intravital fluorescence microscopy and computer-assisted image analysis. Values are means ± s.e.m. ^a^*P*<0.05 versus day 3 within each individual group; *P<0.05 versus LE^+^ fragments.

The differing network morphology between the two groups was associated with marked differences in microhemodynamic parameters. Microvessels of LE^−^ fragments exhibited a diameter of 13 μm at day 3, which slightly decreased to 11 μm at day 14 (Fig. 4A). In the group of LE^+^ fragments, the microvascular diameters rapidly declined from 12 μm at day 3 to 8 μm at day 6 and then remained constant until the end of the experiments (Fig. 4A). Moreover, the centerline red blood cell (RBC) velocity in microvessels of LE^−^ fragments progressively increased from 140 μm/second at day 3 to 350 μm/second at day 14 and was significantly higher compared with that of microvessels in LE^+^ fragments (day 3, 50 μm/second; day 14, 180 μm/second) (Fig. 4B). Accordingly, calculated values of volumetric microvascular blood flow were also markedly higher in LE^−^ fragments throughout the observation period compared with those of LE^+^ fragments (Fig. 4C).

**Fig. 5.**
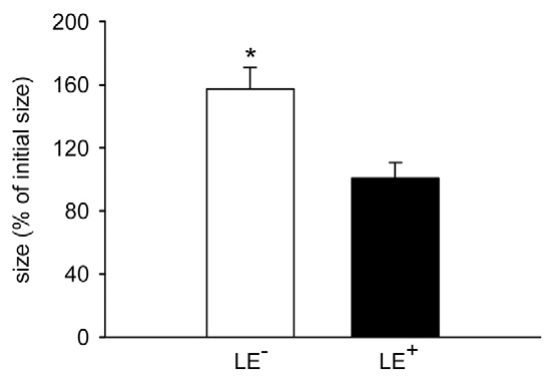
**Size of transplanted endometrial tissue fragments.** Size (as a percentage of initial size) of LE^−^ fragments (white bar, *n*=8) and LE^+^ fragments (black bar, *n*=8) at day 14 after transplantation into dorsal skinfold chambers of C57BL/6 wild-type mice, as assessed by intravital fluorescence microscopy and computer-assisted image analysis. Values are means ± s.e.m. *P<0.05 versus LE^+^ fragments.

Finally, we found that the luminal epithelium markedly affected the growth of LE^+^ fragments. In fact, their size remained constant throughout the observation period (Fig. 5). In contrast, LE^−^ fragments exhibited a significantly larger size at day 14 (Fig. 5).

**Fig. 6.**
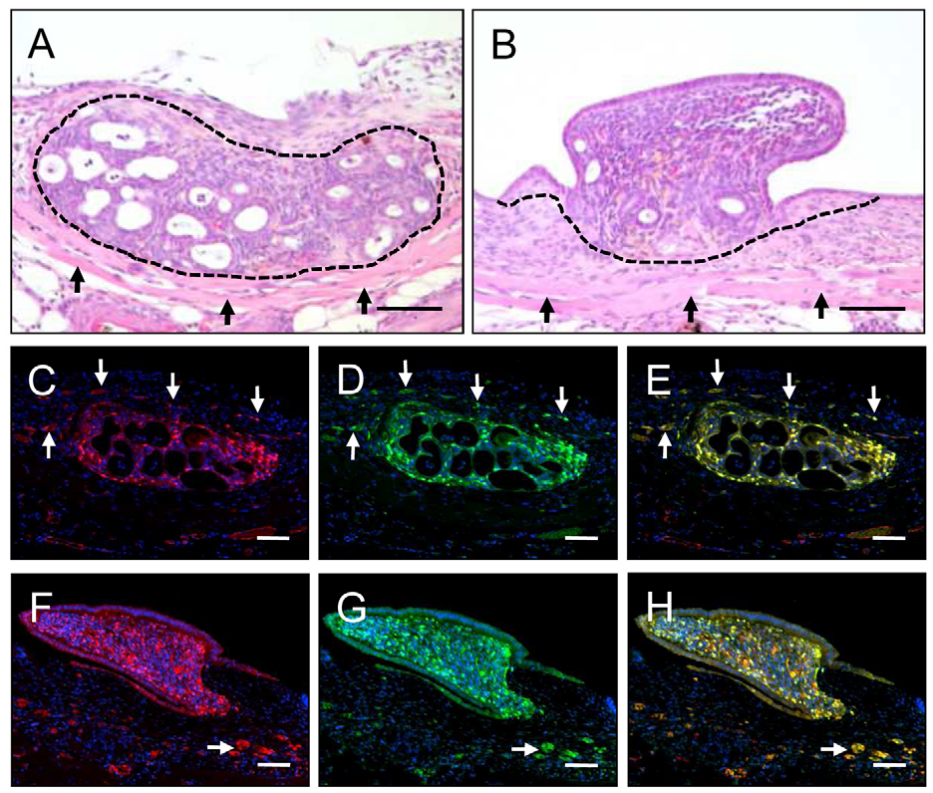
**Histomorphology and blood vessel origin of transplanted endometrial tissue fragments.** (A,B) H&E-stained sections of a LE^−^ fragment (A, borders marked by dashed line) and a LE^+^ fragment (B, borders marked by dashed line) at day 14 after transplantation onto the host striated muscle tissue (arrows) of the dorsal skinfold chamber. The LE^−^ fragment has developed into a typical endometriosis-like lesion with cyst-like dilated endometrial glands, which are surrounded by a well-vascularized stroma. In contrast, the lesion originating from the LE^+^ fragment exhibits a polypoid morphology with less glands and a coverage of luminal epithelium. (C–H) Immunohistochemical analysis of the origin of microvessels within and around a LE^−^ fragment (C–E) and a LE^+^ fragment (F–H) at day 14 after transplantation into the dorsal skinfold chamber of a C57BL/6 wild-type mouse. Histological sections were stained with Hoechst 33342 to identify cell nuclei (C–H, blue), an antibody against CD31 for the detection of endothelial cells (C,F, red) and an antibody against GFP (D,G, green). E displays a merge of C and D, and H a merge of F and G. In contrast to the polypoid lesion originating from the LE^+^ fragment, the endometriosis-like lesion originating from the LE^−^ fragment is surrounded by many GFP^+^ microvessels (arrows). Scale bars: 75 μm (A,B), 70 μm (C–H).

Histological examination of hematoxylin and eosin (H&E)-stained sections of LE^−^ fragments at day 14 after transplantation into the dorsal skinfold chamber revealed typical endometriosis-like lesions with cyst-like dilated endometrial glands, which were surrounded by a well-vascularized stroma (Fig. 6A). In contrast, lesions from LE^+^ fragments exhibited a polypoid morphology with less glands and a coverage of luminal epithelium (Fig. 6B).

**Fig. 7.**
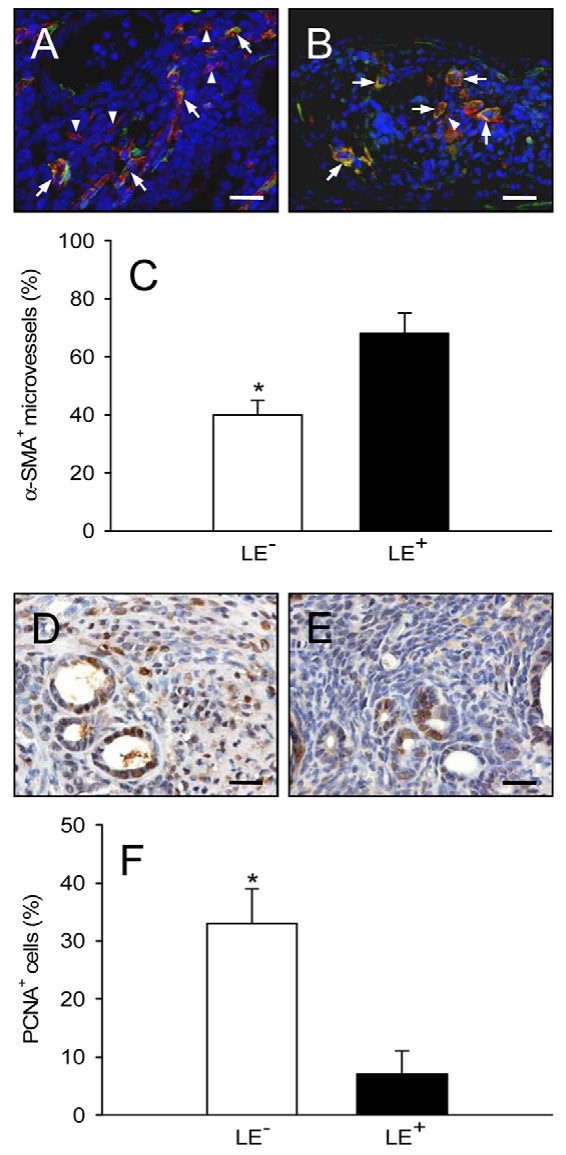
**Vessel maturation and proliferating activity of transplanted endometrial tissue fragments.** (A,B) Immunohistochemical analysis of vessel maturation within a LE^−^ fragment (A) and a LE^+^ fragment (B) at day 14 after transplantation into the dorsal skinfold chamber of a C57BL/6 wild-type mouse. A and B display merges of histological sections stained with Hoechst 33342 to identify cell nuclei (blue), an antibody against CD31 for the detection of microvessels (red) and an antibody against α-SMA (green). Both fragment types contain a mixture of α-SMA^+^ mature microvessels (arrows) and α-SMA^−^ immature microvessels (arrowheads). (C) α-SMA^+^ microvessels (as a percentage of the total number of microvessels) within LE^−^ fragments (white bar, *n*=6) and LE^+^ fragments (black bar, *n*=4), as assessed by immunohistochemical analysis. (D,E) Immunohistochemical detection of PCNA^+^ cells (brown) within a LE^−^ fragment (D) and a LE^+^ fragment (E) at day 14 after transplantation into the dorsal skinfold chamber of a C57BL/6 wild-type mouse. (F) PCNA^+^ cells (as a percentage of the total number of cells) within LE^−^ fragments (white bar, *n*=7) and LE^+^ fragments (black bar, *n*=6), as assessed by immunohistochemical analysis. Values are means ± s.e.m. *P<0.05 versus LE^+^ fragments. Scale bars: 30 μm (A,B), 20 μm (D,E).

More detailed immunohistochemical analyses of both lesion types confirmed our intravital microscopic findings. Endometriosis-like lesions originating from LE^−^ fragments were surrounded by many GFP^+^ microvessels, which grew out of the lesions into the GFP^−^ host tissue of the dorsal skinfold chamber (Fig. 6C–E). In contrast, polypoid lesions originating from LE^+^ fragments only exhibited the outgrowth of a few GFP^+^ microvessels in areas lacking the barrier of the luminal epithelium (Fig. 6F–H). We further assessed the fraction of mature microvessels, which exhibited a perivascular coverage of stabilizing α-smooth muscle actin (SMA)^+^ cells, within the lesions. Both types of lesions contained a mixture of α-SMA^+^ mature microvessels and α-SMA^−^ immature microvessels (Fig. 7A,B). However, the fraction of mature microvessels was significantly lower in the lesions that originated from LE^−^ fragments (Fig. 7C). Finally, in line with our intravital microscopic measurements of lesion sizes, immunohistochemical detection of proliferating cell nuclear antigen (PCNA) revealed a markedly increased proliferating activity within LE^−^ lesions at day 14 compared with LE^+^ lesions (Fig. 7D–F).

## DISCUSSION

Because rodents do not develop spontaneous endometriosis, endometriosis-like lesions are usually induced for research purposes in these species by transplanting endometrial tissue fragments to ectopic sites (Grümmer, 2006). In the present study we demonstrate that this approach is crucially dependent on the cellular composition of the grafts. In fact, we found that the vascularization, morphology and growth of endometriosis-like lesions originating from LE^+^ fragments markedly differ from those of LE^−^ fragments. Whereas LE^−^ fragments develop into typical endometriosis-like lesions with cyst-like dilated endometrial glands and a well-vascularized endometrial stroma, lesions originating from LE^+^ fragments exhibit a polypoid morphology and a reduced blood perfusion after engraftment. This is associated with a decreased growth rate compared with LE^−^ lesions.

Our novel findings are in line with and extend the results of a previous study in which we fixed uterine tissue samples with the luminal epithelium of the endometrium to the peritoneal lining of the abdominal cavity (Körbel et al., 2010). As assessed by high-resolution ultrasound imaging, these grafts presented with lower volumes throughout a 4-week observation period compared with samples that were fixed with the perimetrium to the peritoneum. In addition, they exhibited decreased numbers of proliferating stromal and glandular cells. This can be explained by the natural barrier function of the luminal epithelium (Gipson et al., 2008; Ochiel et al., 2008), which prevented the interaction of the grafts with the surrounding host tissue. Accordingly, we found in the present study that only the areas of LE^+^ fragments that were not covered with luminal epithelium engrafted at the transplantation site.

In our experiments we analyzed the formation of endometriosis-like lesions in the dorsal skinfold chamber model. Although this model does not reflect the natural environment of the peritoneal cavity, it bears in contrast to conventional intraperitoneal endometriosis models the major advantage that the lesions are easily accessible for intravital microscopic analyses (Laschke and Menger, 2007). Therefore, it is possible to study repetitively the morphology and microhemodynamic conditions of newly developing microvascular networks inside endometriosis-like lesions (Laschke et al., 2008; Feng et al., 2012). We herein demonstrate that LE^−^ lesions exhibit an accelerated vascularization and improved blood perfusion compared with LE^+^ lesions. This could be due to the fact that the microvascular networks of LE^−^ lesions developed many interconnections to the surrounding host microvasculature. Such interconnections promote the outflow of blood, which decreases the overall resistance of microvascular networks resulting in elevated blood volume flow and, thus, increased RBC velocities (Less et al., 1997; Yang and Murfee, 2012). In contrast, microvessels did not pass the luminal epithelium of LE^+^ lesions. Accordingly, these lesions were only supplied and drained by a few microvessels at the base of their polypoid structure. In line with these findings, the size of LE^+^ fragments remained constant throughout the observation period, whereas LE^−^ fragments exhibited a high proliferating activity, resulting in a markedly increased size at day 14. This further supports the concept that the engraftment, survival and growth of endometriotic lesions is crucially dependent on an adequate vascularization (Groothuis et al., 2005; Rudzitis-Auth et al., 2013).

The transplantation of GFP^+^ endometrial tissue fragments into dorsal skinfold chambers of GFP^−^ wild-type recipient mice allowed us to study the origin of microvessels within and around the newly developing endometriosis-like lesions. Of interest, we found that both LE^−^ and LE^+^ lesions still exhibited GFP^+^ microvessels at day 14 after transplantation. This proves that these vessels, which originated from the GFP^+^ donor mice, were able to survive the entire isolation, transplantation and engraftment process. Because the lesions did not exhibit an adequate blood perfusion at day 3, hypoxia-induced vasodilation of these microvessels might have caused the observed increase in microvascular diameters compared with later observation time points.

The original microvessels might have also mainly constituted the fraction of mature α-SMA^+^ vessels, which particularly occurred in LE^+^ lesions. In addition, many GFP^+^ microvessels grew outside the LE^−^ lesions into the surrounding host tissue, where they developed interconnections to the host microvasculature, which is termed ‘external inosculation’ (Laschke et al., 2009). We therefore suggest that besides angiogenesis and vasculogenesis (Taylor et al., 2009; Laschke et al., 2011a), the inosculation of pre-existing microvessels within endometrial tissue fragments is an additional important mechanism that contributes to the vascularization of endometriosis-like lesions. Newly formed microvessels of the host tissue could in turn easily grow into the LE^−^ lesions, which could explain the higher fraction of immature α-SMA^−^ vessels within these lesions compared with lesions originating from LE^+^ fragments. In line with this view, LE^−^ lesions also exhibited constantly elevated microvascular diameters over time, which is a typical sign of an immature microvasculature (Laschke et al., 2006).

Endometriotic lesions with a polypoid growth pattern have also been observed under clinical conditions (Mostoufizadeh and Scully, 1980). However, the literature on polypoid endometriosis is limited to a small number of case reports (Parker et al., 2004). Moreover, the etiopathogenesis of this rare type of endometriosis is completely unknown, although postmenopausal age and hyperestrinism have been proposed to be important risk factors (Parker et al., 2004). Based on the observations of the present study, it can be speculated that spread endometrial tissue fragments with intact luminal epithelium contribute to the formation of polypoid lesions. On the other hand, polypoid lesions in endometriosis patients often exhibit hyperplastic or metaplastic glands and sometimes also cytologic atypia (Parker et al., 2004), which indicates that their pathogenesis is much more complex and associated with marked cellular abnormalities.

Taken together, we herein demonstrate that intact luminal epithelium in transplanted endometrial tissue fragments affects their vascularization, growth and morphological development into endometriosis-like lesions in mice. Hence, we suggest that it is of major importance to standardize endometrial grafts in order to increase the validity and reliability of pre-clinical animal studies in endometriosis research. In addition, the present study indicates that various growth patterns of endometriotic lesions might be crucially determined by the initial cellular composition of the ectopic endometrial tissue.

## MATERIALS AND METHODS

### Animals

All experiments were approved by the local governmental animal care committee and were conducted in accordance with the German legislation on protection of animals and the *NIH Guidelines for the Care and Use of Laboratory Animals* (NIH Publication #85-23 Rev. 1985).

For the study, 14- to 16-week-old female C57BL/6-TgN(ACTB-EGFP)1Osb/J mice and corresponding C57BL/6 wild-type mice with a body weight of 22–24 g were used. The animals were housed one per cage within a temperature-controlled environment on a 12 hour/12 hour light-dark cycle and had free access to tap water and standard pellet food (Altromin, Lage, Germany). To exclude discrepancies between individual animals due to different sex hormone levels, estrous cycling was evaluated by cytological analysis of vaginal lavage samples. For this purpose, 15 μl of 0.9% NaCl was carefully pipetted into the vagina and subsequently transferred onto a glass slide for examination under a phase contrast microscope (CH-2; Olympus, Hamburg, Germany). Only those animals that were in the stage of estrus were used as donors and recipients of endometrial tissue fragments for the induction of endometriosis-like lesions.

### Dorsal skinfold chamber model

The dorsal skinfold chamber and its implantation procedure have been described previously in detail (Laschke et al., 2011b). Briefly, C57BL/6 wild-type mice were anesthetized by intraperitoneal (i.p.) injection of ketamine (75 mg/kg body weight; Pharmacia, Erlangen, Germany) and xylazine (15 mg/kg body weight; Rompun, Bayer, Leverkusen, Germany). Subsequently, two symmetrical titanium frames were implanted on the extended dorsal skinfold of the animals, so that they sandwiched the double layer of skin. One layer of skin was completely removed in a circular area of ~15 mm in diameter. The remaining layers consisting of striated skin muscle, subcutaneous tissue and skin were covered with a removable cover glass, which was fixed in one of the titanium frames by means of a snap ring. After the preparation, the animals were allowed to recover from anesthesia and surgery for 48 hours. The animals tolerated the chamber and its preparation well, as indicated by normal feeding and sleeping habits.

### Isolation and transplantation of endometrial tissue fragments

Both uterine horns of anesthetized C57BL/6-TgN(ACTB-EGFP)1Osb/J donor mice were removed and transferred in a plastic Petri dish, filled with 37°C warm Dulbecco’s modified Eagle medium (10% fetal calf serum, 100 U/ml penicillin, 0.1 mg/ml streptomycin; PAA Laboratories, Cölbe, Germany). The uterine horns were opened longitudinally by means of a microscissors under a stereo microscope (M651; Leica Microsystems, Wetzlar, Germany). For the generation of LE^−^ fragments, the myometrium with the underlying perimetrium was carefully removed from one uterine horn. Subsequently, small LE^−^ fragments were excised from the exposed basal endometrium. From the second uterine horn, LE^+^ fragments were isolated from the luminal side of the endometrium.

For transplantation of the endometrial tissue fragments, the cover glass of the dorsal skinfold chamber was removed and the chamber tissue was flushed with 0.9% NaCl. One LE^−^ fragment and one LE^+^ fragment were placed onto the host striated muscle tissue within each chamber, with a maximal distance to each other to exclude their mutual interaction during the engraftment process. Finally, the chamber was closed again with a new cover glass.

### Intravital fluorescence microscopy and microcirculatory analysis

For the *in vivo* microscopy of the transplanted endometrial tissue fragments, the anesthetized mice were fixed on a Plexiglas stage and the observation window of the dorsal skinfold chamber was positioned under a Zeiss Axiotech microscope (Zeiss, Oberkochen, Germany) equipped with a 100-W mercury lamp attached to a filter block for blue, green and ultraviolet light. Due to their GFP signal, the endometrial grafts could easily be detected in the dorsal skinfold chamber in blue light epi-illumination (Fig. 1F–I). After intravenous (i.v.) injection of 0.05 ml 5% fluorescein isothiocyanate (FITC)-labeled dextran 150,000 (Sigma-Aldrich, Taufkirchen, Germany) into the retro-orbital plexus, it was further possible to visualize the newly developing microvessels within the grafts and the surrounding host microvasculature by intravascular staining of the plasma. The microscopic images were recorded by a charge-coupled device video camera (FK6990; Pieper, Schwerte, Germany) and transferred to a DVD system for off-line evaluation. Using 5×, 10× and 20× long-distance objectives (Zeiss), magnifications of 115×, 230× and 460× were achieved on a 14-inch video screen (KV-14CT1E; Sony, Tokyo, Japan).

Quantitative analyses of the microscopic images were performed by means of the software package CapImage (version 8.5; Zeintl, Heidelberg, Germany). They included the determination of the size of the endometrial tissue fragments (as a percentage of their initial size), the functional capillary density of the grafts, i.e. the length of RBC-perfused microvessels per observation area (cm/cm^2^), the diameter of the grafts’ microvessels (μm) and their centerline RBC velocity *V*_RBC_ (μm/second). Volumetric blood flow (VQ, pl/second) of individual microvessels was calculated from *V*_RBC_ and diameter (*d*) for each microvessel as VQ = π (*d*/2)^2^ × *V*_RBC_/*K*, where *K* represents the Baker-Wayland factor (Baker and Wayland, 1974), considering the parabolic velocity profile of blood in microvessels; here, *K*=1.3. In addition, we assessed the final number of microvessels that served as interconnections between the grafts’ microvascular networks and the microvasculature of the surrounding host tissue. For this purpose, the border of the grafts was marked on the intravital fluorescent microscopic images at day 14 after transplantation (Fig. 2A,B, dotted line) and all RBC-perfused microvessels that crossed this border, were counted.

### Experimental protocol

A total of eight LE^+^ fragments and eight LE^−^ fragments from four C57BL/6-TgN(ACTB-EGFP)1Osb/J donor mice were transplanted into the dorsal skinfold chamber of eight C57BL/6 wild-type recipient mice. Intravital fluorescence microscopy was performed on days 0 (day of transplantation), 3, 6, 10 and 14 after transplantation. Microvascular diameters and microhemodynamic parameters were determined by analyzing 10 microvessels per time point. For this purpose, the microvessels were selected randomly inasmuch as those microvessels were chosen that crossed a horizontal line drawn over the center of the video screen. At the end of the *in vivo* experiments (i.e. day 14 after transplantation), the animals were sacrificed with an overdose of the anesthetics and the dorsal skinfold chamber preparations were processed for further histological and immunohistochemical analyses.

### Histology and immunohistochemistry

For light microscopy, formalin-fixed specimens of the dorsal skinfold chamber preparations were embedded in paraffin. Sections of 2 μm thickness were cut and stained with H&E according to standard procedures.

For immunohistochemical detection of GFP^+^ microvessels within and around the transplanted endometrial tissue fragments, paraffin-embedded 2-μm thick sections were stained with a monoclonal rat anti-mouse antibody against CD31 (1:30; Dianova, Hamburg, Germany) to detect endothelial cells and with a goat anti-GFP antibody (1:200; Biomol, Hamburg, Germany) to enhance GFP fluorescence. Secondary antibodies were a goat anti-rat Cy3 antibody (1:50; Dianova) and a biotin-labeled donkey anti-goat antibody (1:15; Jackson ImmunoResearch, Baltimore, MD), which was detected by fluorescein-labeled streptavidin (1:50; Vector Labs, Burlingame, CA). For this purpose, the sections were placed in Coplin jars with 0.05% citraconic anhydride solution (pH 7.4) for 1 hour at 98°C and then incubated overnight at 4°C with the first antibody, followed by the appropriate secondary antibody at 37°C for 2 hours. Additional sections of endometrial tissue fragments were stained with a monoclonal rat anti-mouse antibody against the endothelial cell marker CD31 (1:30; Dianova) and a mouse anti-mouse antibody against α-SMA (1:50; Sigma-Aldrich). A goat anti-rat Cy3 antibody (1:50; Dianova) and a goat anti-mouse Alexa-Fluor-488-conjugated antibody (1:200; Invitrogen, Darmstadt, Germany) served as secondary antibodies. On each section, cell nuclei were stained with Hoechst 33342 (1:500; Sigma-Aldrich) to merge the images exactly. The sections were examined using a BZ-8000 microscopic system (Keyence, Osaka, Japan) and the fraction of mature α-SMA^+^ microvessels was assessed within the fragments (as a percentage of the total number of CD31^+^ microvessels; *n*=4–6).

Immunohistochemical staining of PCNA^+^ cells within the endometrial tissue fragments was performed by a mouse monoclonal anti-PCNA antibody (1:200; Dako Deutschland, Hamburg, Germany) as primary antibody. This was followed by a biotin-labeled goat anti-mouse antibody (1:100; Abcam, Cambridge, UK), which served as secondary antibody. Subsequently, the tissue sections were incubated with avidin-peroxidase (1:50; Sigma-Aldrich). The chromogen used was 3,3′-diaminobenzidine. The sections were counterstained with hemalaun and the fraction of PCNA^+^ cells (as a percentage of the total cell number; *n*=6–7) was assessed by light microscopy (BX60; Olympus, Hamburg, Germany).

### Statistics

Data were first analyzed for normal distribution and equal variance. Differences between LE^+^ fragments and LE^−^ fragments were calculated by the unpaired Student’s *t*-test. To test for time effects in the individual groups, ANOVA for repeated measures was applied. This was followed by the Student-Newman-Keuls test, including the correction of the alpha error according to Bonferroni probabilities to compensate for multiple comparisons (SigmaStat; Jandel Corporation, San Rafael, CA). All values are expressed as means ± s.e.m. Statistical significance was accepted for a value of *P*<0.05.
